# Store-Operated Calcium Entry Is Required for mGluR-Dependent Long Term Depression in Cortical Neurons

**DOI:** 10.3389/fncel.2017.00363

**Published:** 2017-12-14

**Authors:** Paloma González-Sánchez, Araceli del Arco, José A. Esteban, Jorgina Satrústegui

**Affiliations:** ^1^Department of Molecular Biology, Centro de Biología Molecular Severo Ochoa, Consejo Superior de Investigaciones Científicas-Universidad Autónoma de Madrid (CSIC-UAM), Madrid, Spain; ^2^Centro de Investigación Biomédica en Red de Enfermedades Raras (CIBERER), Madrid, Spain; ^3^Instituto de Investigación Sanitaria Fundación Jiménez Díaz (IIS-FJD), Madrid, Spain; ^4^Facultad de Ciencias Ambientales y Bioquímica, Universidad de Castilla la Mancha, Toledo, Spain; ^5^Department of Molecular Neurobiology, Centro de Biología Molecular Severo Ochoa, Consejo Superior de Investigaciones Científicas-Universidad Autónoma de Madrid (CSIC-UAM), Madrid, Spain

**Keywords:** store-operated calcium entry (SOCE), metabotropic glutamate receptors (mGluRs), long-term depression (LTD), calcium signaling, synaptic plasticity

## Abstract

Store-operated calcium entry (SOCE) is a Calcium (Ca^2+^) influx pathway activated by depletion of intracellular stores that occurs in eukaryotic cells. In neurons, the presence and functions of SOCE are still in question. Here, we show evidences for the existence of SOCE in primary mouse cortical neurons. Endoplasmic reticulum (ER)-Ca^2+^ depletion using thapsigargin (Tg) triggered a maintained cytosolic Ca^2+^ increase, which rapidly returned to basal level in the presence of the SOCE blockers 2-Aminoethoxydiphenyl borate (2-APB) and YM-58483. Neural SOCE is also engaged by activation of metabotropic glutamate receptors (mGluRs) with (S)-3,5-dihydroxyphenylglycine (DHPG) (agonist of group I mGluRs), being an essential mechanism to maintain the mGluR-driven Ca^2+^ signal. Activation of group I of mGluRs triggers long-term depression (LTD) in many brain regions, but the underlying mechanism and, specifically, the necessity of Ca^2+^ increase in the postsynaptic neuron is controversial. In primary cortical neurons, we now show that the inhibition of Ca^2+^ influx through SOCE impaired DHPG-LTD, pointing out a key function of calcium and SOCE in synaptic plasticity.

## Introduction

Calcium (Ca^2+^) is a universal second messenger that regulates numerous cellular processes of all eukaryotic cells, including metabolism, muscle contraction, exocytosis, transcription of numerous genes or programmed cell death (Brini et al., 2013). In neurons, it also plays a critical role in the transmission of synaptic information by controlling neurotransmitter release (Südhof, 2012). In addition, calcium is the intracellular messenger for many forms of activity-dependent synaptic plasticity acting both in the presynaptic and/or postsynaptic neuron (Zucker, 1999; Cavazzini et al., 2005). These roles are performed thanks to abundant and specific ligand- and voltage-gated Ca^2+^ channels which exert a tight control on Ca^2+^ dynamics (Grienberger and Konnerth, 2012; Brini et al., 2014). Ca^2+^ stores also have a relevant role in the regulation of the intracellular Ca^2+^ concentration (Verkhratsky, 2005).

Ca^2+^ homeostasis of the endoplasmic reticulum (ER) is controlled by several and well-orchestrated mechanisms (Stutzmann and Mattson, 2011). In mammals, the release of Ca^2+^ from ER is sensed by the stromal interaction molecule (STIM) proteins, STIM1 and STIM2 (Liou et al., 2005; Roos et al., 2005). After a decrease in ER-Ca^2+^ levels, STIM proteins oligomerize and migrate to the ER-plasma membrane (PM) junctions, where they interact with Orai calcium channels, Orai1, Orai2 or Orai3 (Feske et al., 2005; Gwack et al., 2007) to allow Ca^2+^ influx into the cytosol. This mechanism is called store-operated calcium entry (SOCE; Putney, 1986), and it has been extensively studied in non-excitable cells, for which SOCE acts as a principal Ca^2+^ entry pathway (Parekh and Putney, 2005; Prakriya and Lewis, 2015). Conversely, the relevance of neuronal SOCE is being debated (Lu and Fivaz, 2016). In the last few years, SOCE activity has been observed in hippocampal (Emptage et al., 2001; Baba et al., 2003; Kann et al., 2012; Sun et al., 2014; Samtleben et al., 2015), cortical (Berna-Erro et al., 2009; Klejman et al., 2009; Gruszczynska-Biegala et al., 2011), cerebellar (Baba et al., 2003; Hartmann et al., 2014), sensory (Gemes et al., 2011) and dorsal horn neurons (Xia et al., 2014). Moreover, two independent groups found that STIM1 directly modulates depolarization-induced opening of the voltage-gated Ca^2+^ channel Ca_V_1.2 (Park et al., 2010; Wang et al., 2010). Neuronal SOCE is thought to perform different roles: the refilling of ER-Ca^2+^, which is continuously emptying at rest (Samtleben et al., 2015), regulation of neuronal gene expression (Lalonde et al., 2014), and the maturation and maintenance of dendritic spines (Sun et al., 2014; Korkotian et al., 2017). An impairment in SOCE function related to a mislocalization of mitochondria close to Ca^2+^ entry sites has been shown to occur in Charcot-Marie-Tooth disease related to recessive mutations in ganglioside-induced differentiation associated protein 1 (GDAP1; Pla-Martín et al., 2013; González-Sánchez et al., 2017).

Recently, several studies have suggested a relevant role for ER-Ca^2+^ stores and SOCE in synaptic plasticity (Bardo et al., 2006; Majewski and Kuznicki, 2015; Moccia et al., 2015; Segal and Korkotian, 2016). SOCE has been shown to control neuronal Ca^2+^ dynamics during synaptic activity in different neurons (Moccia et al., 2015). A direct link between SOCE and AMPA receptor-dependent Ca^2+^ signal has been proposed (Gruszczynska-Biegala et al., 2016). A mechanism that links activation of postsynaptic NMDA receptors and L-type Ca^2+^ channels has been linked to signaling by the ER-Ca^2+^ sensor STIM1 (Dittmer et al., 2017). STIM1 has also been proposed to be a key regulator of Ca^2+^ signaling downstream metabotropic glutamate receptor (mGluR) stimulation (Hartmann et al., 2014; Hou et al., 2015).

Activation of group I of mGluRs (mGluRs I) using electrical or pharmacological stimulation ((S)-3,5-dihydroxyphenylglycine (DHPG)) leads to long-term depression (LTD) in many brain regions (Lüscher and Huber, 2010). mGluRs I are canonically coupled to Gα_q/11_ and the activation of phospholipase Cβ (PLC), inositol triphosphate (IP_3_) generation, release of Ca^2+^ from intracellular stores, and protein kinase C (PKC) activation (Abe et al., 1992; Aramori and Nakanishi, 1992). However, depending on the induction protocol and the brain region, there are differences in the involvement of the PLC pathway and postsynaptic Ca^2+^ rise in mGluR-LTD (Gladding et al., 2009; Lüscher and Huber, 2010).

Although LTD takes place in many brain regions, most studies have focused on hippocampal neurons. The aim of the present study was to investigate the role of SOCE in cortical neuron LTD, specifically upon mGluR stimulation.

## Materials and Methods

### Animals

Wild-type mice with a mixed C57BL6/Sv129 genetic background were used. Mice were housed in a humidity- and temperature-controlled room on a 12 h light/dark cycle, receiving water and food *ad libitum*. All the experimental protocols performed in this study were performed in accordance with procedures approved in the Directive 86/609/EEC of the European Union and with approval of the Institutional Ethical Committee of the Center of Molecular Biology Severo Ochoa and Universidad Autonoma de Madrid (CEEA-CBMSO-23/159) and Area de Protection Animal Comunidad de Madrid (PROEX 352/15). All efforts were made to minimize animal suffering.

### Primary Neuronal Culture

Cortical neuronal cultures were prepared from E15 to E16 mouse embryos as described earlier (Ramos et al., 2003; Pardo et al., 2006). Neurons were plated at a density of 5 × 10^5^ cells/cm^2^ on poly-L-lysine and laminin-coated pretreated glass coverslips in Neurobasal medium supplemented with 2% B27, 1% glutamax (all from Gibco Invitrogen, Carlsbad, CA, USA) and 100 mg/ml penicillin–streptomycin. On the fifth day *in vitro* (DIV) half of the plating medium was removed from each well and replenished with BrainPhys medium (Bardy et al., 2015; Stem cell Technologies, Seattle, WA, USA) supplemented with 2% B27 and 100 mg/ml penicillin–streptomycin. Neurons represented >80% of the total cell population (Ramos et al., 2003; Pardo et al., 2006). The cultures were maintained at 37°C in a humidified atmosphere of 5% CO_2_. The culture medium was partially replaced every other day. Cultures were used for experimentation between 9 and 11 DIV. BrainPhys was chosen as culture medium for these studies because it has an inorganic salt concentration, glucose level and osmolarity similar to those reported for the brain, and neurons grown in this culture medium show high viability and maintain synaptic network activity during long time in culture (Bardy et al., 2015), i.e., conditions adequate for the study of possible roles of SOCE in synaptic activity. However, we have verified that SOCE activity in cortical neurons was independent of the culture medium, and we have observed a similar and consistent Ca^2+^ entry after ER-Ca^2+^ discharge using thapsigargin (Tg) in cortical neurons cultured in both BrainPhys and Neurobasal medium.

### Measurement of Cytosolic Ca^2+^ Signals

Cytosolic calcium imaging with Fura-2 was performed as described by Ruiz et al. (1998). Neurons were plated at 2 × 10^5^ cells/well onto 12 mm round coverslips. Cells were loaded with Fura-2AM by incubation in 2.5 mM D-glucose Ca^2+^-free HCSS with 5 μM Fura-2AM and 50 μM pluronic F.127 acid (both from Molecular Probes, Invitrogen, Carlsbad, CA, USA), for 30 min at 37 °C, and rinsed with HCSS 2 mM CaCl_2_, for 30 min. Fluorescence (emission 510 nm) ratio of Ca^2+^-free (F380) to Ca^2+^-bound probe (F340) was analyzed using Aquacosmos 2.5 software (Hamamatsu) and Metafluor for Leica developed by Metamorph (Universal Imaging). Regions of interest (ROIs) were selected covering single cells. SOCE analysis was performed using a 2 mM CaCl_2_ and 1 μM tetrodotoxin (TTX, Tocris Bioscience, Bristol, UK) medium, ER-Ca^2+^ was depleted using 1 μM of Tg (Alomone Labs, Jerusalem, Israel). Drugs used to study the influence of other neuronal Ca^2+^ channels were: 10 μM 6-cyano-7-nitroquinoxaline-2,3-dione (CNQX), 10 μM MK-801 ((5S,10R)–(+)-5-Methyl-10,11-dihydro-5H-dibenzo[a,d]cyclohepten-5,10-imine hydrogen maleate), 50 μM NiCl_2_ (all from Sigma-Aldrich, St. Louis, MO, USA) and 5 μM MPEP (2-Methyl-6-(phenylethynyl) pyridine) (Tocris Bioscience, Bristol, UK). mGluR analysis was performed in a 2 mM CaCl_2_ and 1 μM TTX medium, where 200 μM DHPG (Tocris Bioscience, Bristol, UK) was added in absence or presence of the SOCE blockers 10 μM YM-58483 (Tocris Bioscience, Bristol, UK) or 50 μM 2-Aminoethoxydiphenyl borate (2-APB; Sigma-Aldrich, St. Louis, MO, USA).

### Quantitative Real Time PCR (qRT-PCR)

Levels of messenger RNA (mRNA) in neurons were determined by quantitative real time PCR (qRT-PCR). Specific intron-spanning primers for PCR amplification of the SOCE genes: Orai1, 2 and 3, Stim1 and 2, Trpc1, 2, 3, 4, 5, 6 and 7, were designed using Universal Probe Library web^1^ (Roche, Basel, Switzerland). RNA extraction from 8 to 9 DIV neurons was done with RNeasy mini kit (QIAGEN, Hilden, Germany) following the manufacturer’s instructions. One microgram of total RNA was subjected to first strand cDNA synthesis using random hexamers with Avian Myeloblastosis Virus (AMV) reverse transcriptase (Promega, Madison, WI, USA) and quantified using the spectrophotometer NanoDrop 1000 (ThermoFisher, Waltham, MA, USA).

For qPCR, 2.5 ng of the cDNA synthesized was amplified using the ABI Prism 7900HT real-time PCR System (Applied Biosystems, Foster City, CA, USA). The amplification protocol was: hot start (10 min 95°C) and 40 amplification cycles (15 s 95°C, 1 min 60°C). All reactions were carried out in triplicate. Primer sequences for cDNA analysis by qRT-PCR were as follows (5′-3′): Orai1 forward tacttaagccgcgccaag; Orai1 reverse acttccaccatcgctacca; Orai2 forward cacagacgctagccacgag; Orai2 reverse atgggcacattgagctctg; Orai3 forward cacatctgctctgctgtcg; Orai3 reverse aggcctggtgggtattcat; Stim1 forward cagggactgtactgaagatgaca; Stim1 reverse aggtgattatgccgagtcaag; Stim2 forward gagggcgcagagtgtgag; Stim2 reverse tttagagccatgcggacct; Trpc1 forward tgtggttgtgattgtgctga; Trpc1 reverse tccattctttatcctcatgatttg; Trpc2 forward cccatcgggacctttacc; Trpc2 reverse tcgaaggcggtaggacac; Trpc3 forward ttaattatggtctgggttcttgg; Trpc3 reverse tccacaactgcacgatgtact; Trpc4 forward aaggaagccagaaagcttcg; Trpc4 reverse ccaggttcctcatcacctct; Trpc5 forward ggcgatgcattactctacgc; Trpc5 reverse atcatcagcgtgggaacct; Trpc6 forward tactggtgtgctccttgcag; Trpc6 reverse caaacttcatgaacggtcctc; Trpc7 forward aacgatgaagtcaatgaaggtg; Trpc7 reverse ccagctctcctgtagcctga. We performed an absolute quantification using linearized plasmids containing the cDNA fragments and the standard curve method. For quantification of the PCR products, we used the fluorescent dye SYBR-Green (SYBR^®^ Green, Biorad, Hercules, CA, USA). The parameter analyzed was the quantification cycle (Cq). A standard curve of each amplicon (from 10 to 10^8^ copies) was used and the number of copies of SOCE genes was obtained by extrapolation. The PCR efficiency was calculated for each pair of specific primers and was applied where appropriate.

### Western Blot

Cortical neurons were plated at a density of 1 × 10^6^ cells/well on poly-L-lysine and laminin-coated pretreated six wells plates. Cells were collected with a scrapper into a homogenization buffer (250 mM Sucrose, 20 mM Hepes, 10 mM KCl, 1.5 mM MgCl_2_, 1 mM EDTA, 1 mM EGTA, 1 mM DTT, Complete protease inhibitor cocktail mini-EDTA free, (Roche, Basel, Switzerland) and adjusted to pH 7.4). The samples were homogenized by sonication, and aliquots of 30 μg protein (Bradford assay) were separated by SDS-PAGE and transferred to nitrocellulose membranes. Primary antibodies used were α-ORAI1 (1:200) rabbit polyclonal, α-ORAI2 (1:200) rabbit polyclonal, α-STIM2 (1:500) rabbit polyclonal, α-TRPC1 (1:500) rabbit polyclonal, α-TRPC4 (1:500) rabbit polyclonal, (all from Alomone Labs, Jerusalem, Israel) and α−βActin (1:5000) mouse monoclonal (Sigma-Aldrich, St. Louis, MO, USA).

### Electrophysiological Recordings and mEPSC Analysis

Voltage-clamp whole-cell recordings were performed in DIV 10–11 cortical neurons. Neurons at 37°C were continuously perfused with a recording medium containing (in mM) 120 NaCl, 0.8 MgCl_2_, 5.4 KCl, 25 HEPES, 2.5 D-glucose, 2 CaCl_2_ and supplemented with 1 μM TTX to prevent action potential-evoked responses; pH was adjusted to 7.4. Patch electrodes with resistances between 4–6 MΩ were filled with an internal solution containing (in mM) 115 K gluconate, 20 KCl, 10 HEPES, 2 MgCl_2_, 4 Na_2_-ATP, 0.3 Na_3_-GTP, pH adjusted to 7.3 and osmolarity ~290 mOsm. Neurons were voltage-clamped at −60 mV. Electrophysiological recordings and data acquisition were carried out with a Multiclamp 700B amplifier and Clampfit 10.7 software (Molecular Devices, Sunnyvale, CA, USA). Miniature excitatory postsynaptic currents (mEPSC) were detected and analyzed using the Clampfit EventDetection module. Only events with an amplitude >8 pA, an exponential decay and a monotonic rising phase, which could be clearly discriminated from the background noise, were considered as mEPSCs. mEPSC amplitude and frequency were compared during a 2-min baseline period and in 2-min windows 15 min after 200 μM DHPG application (5 min). Decay time constants (τ) were calculated from single exponential fits of averaged mEPSC traces.

### Statistical Analysis

Statistical analyses were performed using STATISTICA software, version 7 (StatSoft). Shapiro-Wilk test was applied to determine the distribution of the data. In normal distributions, one-way or two-way ANOVA test, following of a *post hoc* Bonferroni test were applied. In non-normal distributions (mEPSC parameters) Wilcoxon matched paired test was applied. Significance was *p < 0.05, **p < 0.01, ***p < 0.001. All data are expressed as the mean ± SEM.

## Results

### Store-Operated Ca^2+^ Entry Is Induced in Cortical Neurons after Store Depletion

**Figure 1:**
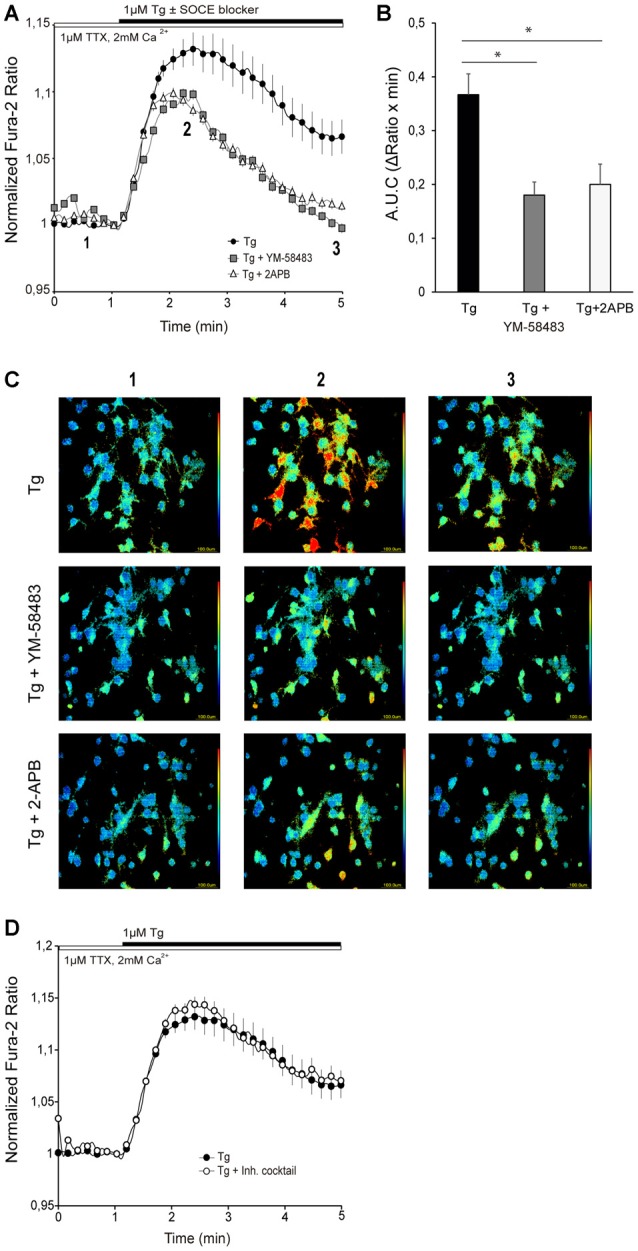
Store-operated calcium entry (SOCE) activity in primary cortical neurons. **(A)** Fura-2 [Ca^2+^]_i_ signals in cortical neurons in HCSS medium containing 2 mM CaCl_2_ and 1 μM tetrodotoxin (TTX), upon addition of 1 μM thapsigargin (Tg) ± 10 μM YM-58483 or 50 μM (2-Aminoethoxydiphenyl borate (2-APB) were indicated. The numbers refer to the frames reported in the **(C)**. **(B)** Quantification of area under SOCE curve (A.U.C, ΔRatio·min). Data were obtained from three independent experiments (*n* = 3), and in each one a total of 25–30 cells were analyzed. **(C)** Representative color-coded Fura-2 [Ca^2+^]_i_ ratio images from basal situation (1), immediately after Tg ± SOCE blockers addition (2), and the end of the measurement. Pseudo-color ratio images were obtained using Aquacosmos 2.5 software (Hamamatsu). **(D)** Fura-2 [Ca^2+^]_i_ signals in cortical neurons in HCSS medium containing 2 mM CaCl_2_ and 1 μM TTX ± inhibitory cocktail (10 μM CNQX (6-cyano-7-nitroquinoxaline-2,3-dione), 10 μM MK-801, 5 μM MPEP, 50 μM NiCl_2_), upon addition of 1 μM Tg. Data were obtained from three independent experiments (*n* = 3), and in each one a total of 25–30 cells were analyzed. All data are normalized to the initial values and are expressed as mean ± SEM. Means were compared using one-way ANOVA, *p < 0.05, *post hoc* Bonferroni test.

To assess the presence of SOCE pathway in primary cortical neurons, we depleted ER stores using 1 μM Tg, the sarco/endoplasmatic reticulum Ca^2+^ ATPase (SERCA) inhibitor, in a medium with 1 μM TTX, in order to prevent neuronal activity. Tg application resulted in a sustained increase in cytosolic calcium (Figure 1A, black circles) that lasted for several minutes. To determine whether Ca^2+^ entering the cell through SOC channels was contributing to this cytosolic Ca^2+^ rise, we applied different widely used SOCE blockers along with Tg: 2-APB and YM-58483 (also called BTP2; Xia et al., 2014; Prakriya and Lewis, 2015). YM-58483 is highly specific and has been shown not to interfere with voltage-gated calcium channels (VGCC; Xia et al., 2014), and 2-APB is a commonly used bimodal SOCE modulator which decreases SOCE activity at high concentrations and also inhibits IP_3_ signaling (Prakriya and Lewis, 2015). In neurons, it is known that ER Ca^2+^ is continuously being released and replenished and consequently SOCE is active even in resting conditions (Samtleben et al., 2015). To avoid a drop in ER calcium caused by SOCE inhibition, the SOCE blockers were not preincubated but applied together with Tg. In the presence of 10 μM YM-58483 or 50 μM 2-APB, the Tg-induced cytosolic Ca^2+^ signal was strongly attenuated, with a rapid decrease 1–2 min after onset (Figures 1A–C). This result is consistent with a Ca^2+^ entry through SOC channel upon depletion of Ca^2+^ ER stores with Tg.

In addition, to test the possibility that neuronal activity contributed to this calcium signal, cytosolic Ca^2+^ levels were recorded in the presence of an inhibitor cocktail containing 10 μM CNQX (an AMPA/Kainate receptor inhibitor), 10 μM MK-801 (NMDA receptor inhibitor) and 5 μM MPEP (a mGluR inhibitor) to block excitatory action of ionotropic and metabotropic glutamate receptors, 10 μM TTX to block voltage-gated sodium channels, and 50 μM NiCl_2_ to block voltage-activated T-type calcium channels (the inhibition cocktail was present during the whole experiment). Figure 1D shows that the cytosolic Ca^2+^ increase triggered by emptying ER-Ca^2+^ was similar both in the absence and the presence of the inhibitory cocktail. This result contrasts with those recently reported in rat cortical neurons using a different SOCE induction protocol, in which AMPA receptors appeared to contribute to SOC mediated Ca^2+^ entry (Gruszczynska-Biegala et al., 2016). This difference could arise from the protocols used to measure SOCE activity in neurons. In this study, ER-Ca^2+^ depletion was triggered in a medium containing physiological calcium concentration, while Gruszczynska-Biegala et al. (2016) employed the Ca^2+^ addback protocol, in which addition of Ca^2+^ to the external medium may activate neuronal Ca^2+^ channels by changing the membrane excitability as described elsewhere (Lu and Fivaz, 2016).

### SOC Channel Proteins Are Expressed in Mouse Cortical Neurons

**Figure 2:**
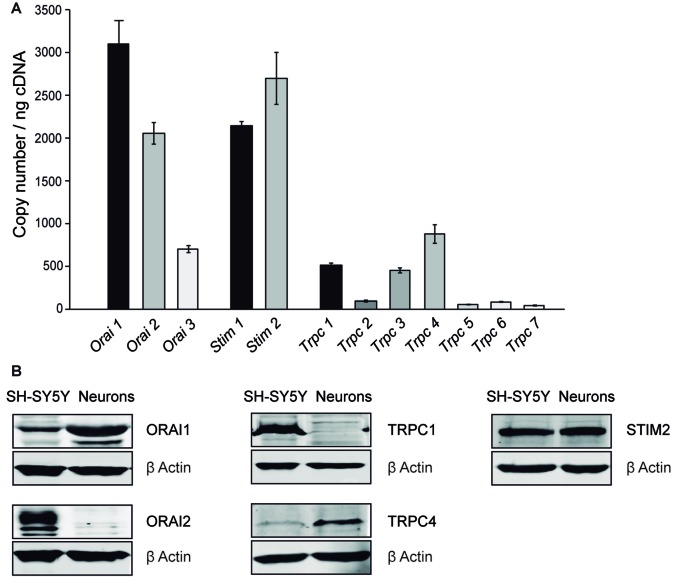
SOC channel proteins in mouse cortical neurons.** (A)** Real time PCR (RT-PCR) of DNA complementary from primary cortical neurons obtained from E-15 wild-type mouse embryo with *Orai* 1–3, *Stim* 1, 2 and *Trpc* 1–7 specific primers. The experiment was repeated four times with similar results. **(B)** Western Blot analysis of ORAI 1, ORAI 2, STIM 2, TRPC 1 and TRPC 4 levels. Protein extracts were obtained from SH-SY5Y cells and 8–9 day *in vitro* (DIV) cortical neurons. Primary antibodies used were α-Orai1, α-Orai2, α-Trpc1, α-Trpc4, α-Stim2 and α -β Actin as a control.

We have studied the different candidate proteins forming SOC channels in other cell types, Orai and Trpc families as calcium channels located in PM and Stim family as calcium sensor in ER-membrane. We first investigated the mRNA levels of the different isoforms of the SOCE components, Orai, Stim and Trpc families, by RT-qPCR in 8–9 DIV cultured cortical neurons from four independent embryos. The profiles obtained for each embryo were quite similar to one another and we found that the isoforms forming the pore channel with highest mRNA levels were Orai1, Orai2, Trpc1 and Trpc4, while Stim1 and Stim2 mRNA levels were similar (Figure 2A). Then, protein levels of the SOCE components with highest mRNA levels were investigated by Western Blot. As positive controls, we used the human neuroblastoma SH-SY5Y cell line, which expresses these proteins (Olianas et al., 2014). We observed a band at 50 kDa with the ORAI1 antibody, a single band at 110 kDa with the TRPC4 antibody and a band at 90 kDa with the STIM2 antibody, but not bands corresponding to ORAI2 and TRPC1 proteins (Figure 2B). These results show that in cultured cortical neurons, ORAI1, STIM2 and TRPC4 were the predominant members of their respective families, in agreement with previous reports in cortical and other neurons of the central nervous system (CNS; Berna-Erro et al., 2009; Steinbeck et al., 2011; Korkotian et al., 2017). STIM1 proteins are also present in mouse cortical neurons (Hou et al., 2015; Guner et al., 2017), but STIM2 has been described as predominant isoform in cortex and hippocampus (Berna-Erro et al., 2009; Steinbeck et al., 2011), while STIM1 protein expression is higher in cerebellum (Lein et al., 2007; Hartmann et al., 2014; Kraft, 2015). Therefore, ORAI1, TRPC4 and STIM1 and 2, are probably responsible for SOC entry in mouse cortical neurons.

### SOCE Is Involved in mGluR-Driven Ca^2+^ Signals

After determining that ER-Ca^2+^ depletion in neurons triggers Ca^2+^ influx into the cytosol by SOCE, we sought to study the activation of this pathway by physiological agonists, particularly glutamate. Activation of group I mGluRs (mGluRs I), formed by mGluR1 and 5, is coupled to a variety of signaling pathways. The Gα_q_/Gα_11_ protein/PLC/inositol-3,4,5-triphosphate (IP_3_) signal cascades have been considered as the canonical pathway, which leads to Ca^2+^ release from ER and activation of PKC. The decrease of ER-Ca^2+^ levels could activate SOCE in the PM and play a role in mGluR-driven Ca^2+^ signaling.

**Figure 3:**
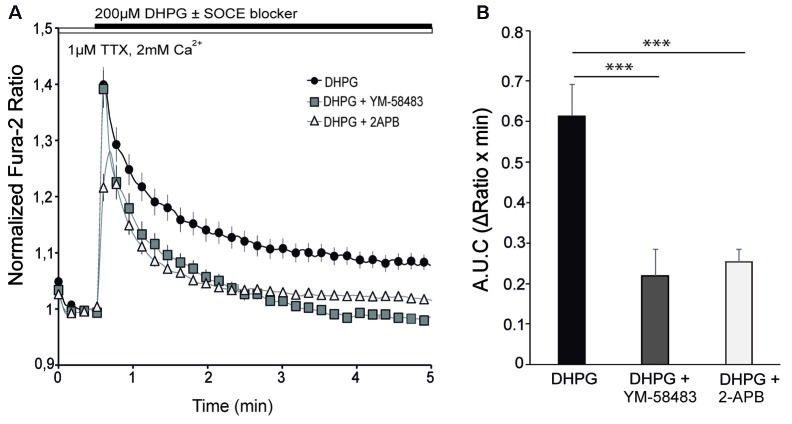
SOCE is involved in metabotropic glutamate receptors (mGluRs)-driven Ca^2+^ signal. **(A)** Fura-2 [Ca^2+^]_i_ signals in cortical neurons in HCSS medium containing 2 mM CaCl_2_ and 1 μM tetrodotoxin (TTX) upon addition of 200 μM (S)-3,5-dihydroxyphenylglycine (DHPG) ± 10 μM YM-58483 or 50 μM 2-APB were indicated. **(B)** Quantification of area under the curve (ΔRatio·min) after DHPG ± YM-58483 or 2-APB addition. Data were obtained from three independent experiments (*n* = 3), and in each one a total of 25–30 cells were analyzed. All data are expressed as mean ± SEM and means were compared using one-way ANOVA, ***p < 0.001, *post hoc* Bonferroni test.

The experiments were performed at 10–12 DIV in a medium with 1 μM TTX in order to eliminate spontaneous activity. Application of 200 μM DHPG, a selective agonist of group I mGluRs, evoked a rapid increase in cytosolic Ca^2+^ followed by a more moderate but sustained Ca^2+^ signal that did not reach baseline during the recording period (Figure 3A, black circles). To determine the contribution of SOCE to the Ca^2+^ response to DHPG, we applied the SOCE blockers together with the agonist of mGluR I, as in previous experiments with Tg (Figure 1). Interestingly, we found that the cytosolic Ca^2+^ transient rapidly returned to baseline when 10 μM YM-58483 or 50 μM 2-APB were added together with DHPG (Figures 3A,B). This result indicates that SOCE is responsible for the sustained increase in Ca^2+^ levels above baseline in response to DHPG. On the other hand, the presence of YM-58483 did not affect the initial Ca^2+^ peak evoked by mGluRs, suggesting that SOC channels open secondarily to mGluRs activation and that the first peak involves the ER-Ca^2+^ mobilization by PLC pathway. In contrast, the addition of 2-APB decreased the initial Ca^2+^ peak, consistent with the effect of this compound on IP_3_ signaling (Prakriya and Lewis, 2015).

### SOCE Inhibition Impairs DHPG-LTD

**Figure 4:**
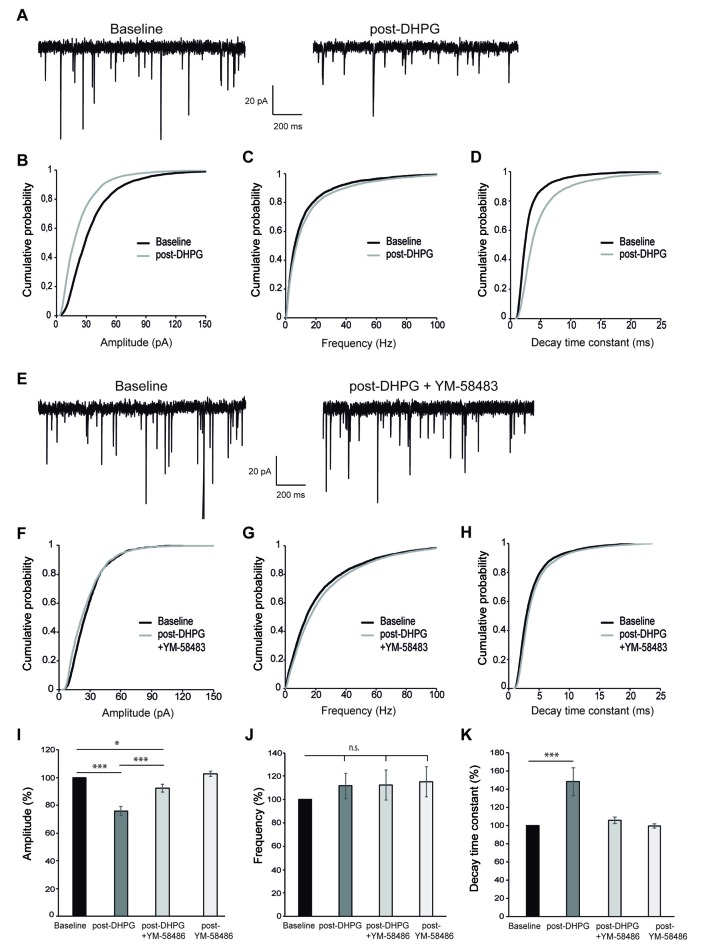
DHPG-long-term depression (LTD) is impaired by SOCE inhibition. **(A)** Representative miniature excitatory postsynaptic current (mEPSC) recordings from a cortical neuron before (left, “baseline”) and 15 min after 200 μM DHPG application (right, “post-DHPG”). **(B–D)** Cumulative probability graphs for amplitude **(B)**, frequency **(C)** and decay time constant **(D)** of mEPSCs, showing baseline (2 min) and a 2 min interval beginning 15 min after DHPG application. **(E)** Representative mEPSC recordings from a cortical neuron before and 15 min after 200 μM DHPG + 10 μM YM-58483 application. **(F–H)** Cumulative probability graphs for amplitude **(F)**, frequency **(G)** and decay time constant **(H)** of mEPSCs, showing baseline (2 min) and a 2 min interval beginning 15 min after DHPG + YM-58483 application. **(I–K)** Mean of mEPSCs amplitude (%) **(I)**, frequency (%) **(J)** and decay time constant **(K)** comparing baseline (100%) to values obtained 15 min after application of 200 μM DHPG, 200 μM DHPG + 10 μM YM-58483 and 10 μM YM-58483 respectively. Data are expressed as mean ± SEM and were obtained from 14 independent experiments for results of DHPG addition, 13 for those of DHPG addition along with YM-58483 and six for those of YM-58483 application alone. Due to non-normal distribution of mEPSCs parameters, statistics were performed using the Wilcoxon matched pairs test, *p < 0.05, ***p < 0.001.

Finally, we wished to determine whether SOCE-dependent calcium signal in response to mGluR activation had physiological consequences for neuronal function. Stimulation of group I mGluRs can lead to a decrease in synaptic strength at many synapses, referred to as long-term depression (mGluR-LTD; Lüscher and Huber, 2010). This form of LTD can be induced by the application of the group I selective mGluR agonist DHPG (so called DHPG-LTD; Xiao et al., 2001; Jo et al., 2008), but the underlying molecular mechanism is still poorly understood and appears to depend on the specific neuronal type. Having shown that SOCE was activated after stimulation of group I mGluRs and was involved in the maintenance of cytosolic Ca^2+^ signal, we investigated whether the activation of SOCE may be involved in DHPG-LTD in cortical neurons. To this end, we first examined the effect of DHPG on synaptic transmission in primary cortical neurons. We found that bath application of 200 μM DHPG (5 min) caused a long lasting reduction of the mEPSC amplitude at 15 min after mGluR stimulation (Figures 4A,B,I). In contrast, we did not find any significant effect on the frequency of mEPSCs (Figures 4A,C,J), suggesting that this type of LTD is predominantly expressed via a postsynaptic mechanism. This interpretation is also consistent with an increase in the decay time constant of mEPSCs after DHPG application (Figures 4D,K). To study the contribution of SOCE to this form of synaptic plasticity, we evaluated the effect of the SOCE blocker YM-58483 (10 μM) applied together with DHPG. Interestingly, the long lasting decrease of mEPSCs amplitude was significantly attenuated (7.7 ± 2.9%; Figures 4E,F,I), as well as the effect on mEPSC kinetics (Figures 4H,K). The frequency of mEPSCs was not affected by DHPG with or without YM-58483 (Figures 4G,J). Importantly, bath application of 10 μM YM-58483 did not have any effect on its own on the amplitude, frequency or kinetics of mEPSCs (Figures 4I–K). All these results strongly suggest that the calcium influx via SOCE after group I mGluRs stimulation is a necessary step for the development of DHPG-LTD in cortical neurons.

## Discussion

SOCE is the main mechanism to replenish intracellular calcium stores in non-excitable cells but its existence in neurons is under debate (Putney, 2003; Lu and Fivaz, 2016), as neurons exhibit other major Ca^2+^ influx pathways through VGCCs and ionotropic glutamate receptors (Grienberger and Konnerth, 2012). Whereas Ca^2+^ entry through SOC channels has been reported in different neuronal populations (Emptage et al., 2001; Berna-Erro et al., 2009; Gemes et al., 2011; Alkhani et al., 2014; Sun et al., 2014; Xia et al., 2014), other groups did not find a clear SOCE response in neurons (Park et al., 2010; Garcia-Alvarez et al., 2015a). One of the possible reasons for this discrepancy could stem from the commonly used Ca^2+^ addback protocol to study SOCE, in which neuronal stores are emptied in a Ca^2+^-free medium, and then Ca^2+^ is added to the external medium. Neurons express multiple Ca^2+^ channels that can be activated in response to changes in extracellular calcium. In the present study, we investigated the existence of SOCE in cortical neurons in relatively mature state, in a medium containing physiological calcium concentrations. Our results clearly showed a small but maintained increase in cytosolic Ca^2+^ after Tg application, which rapidly returned to basal levels in the presence of SOCE blockers, and was unaltered after a cocktail of channel/receptor inhibitors, i.e., all conditions consistent with a bona fide Tg-induced SOCE. The analysis of the molecular players of SOCE in mouse cortical neurons, showed that STIM2 and probably STIM1 are highly expressed at the mRNA and protein levels, in agreement with previous findings (Berna-Erro et al., 2009; Gruszczynska-Biegala et al., 2011; Guner et al., 2017). In the PM, ORAI1 seems to form the store-operated pore, and to a lesser extent, TRPC4, as reported for cortical and hippocampal neurons (Klejman et al., 2009; Korkotian et al., 2017).

A second finding of this study was that a physiological stimulus, group I mGluR activation, induces SOCE in mouse cortical neurons. Group I metabotropic receptors are linked to activation of PLC, IP_3_ generation and release of Ca^2+^ from intracellular stores (Niswender and Conn, 2010). Here, we show that store depletion after mGluR I stimulation using the specific agonist DHPG activates SOCE in primary cortical neurons, and SOCE was necessary to maintain the mGluR-driven cytosolic Ca^2+^ signal. This result is in agreement with a previous report in which stimulation of mGluR I triggered STIM1 oligomerization and migration to PM (Ng et al., 2011), the first step needed in SOCE induction (Lewis, 2007). In fact, more recent findings point to a central role of STIM proteins as one of the intracellular links between mGluR I and its downstream effectors (Hartmann et al., 2014; Hou et al., 2015).

mGluRs are widely distributed throughout the CNS and play relevant roles for synaptic transmission, activity-dependent synaptic plasticity and higher cognitive functions (Niswender and Conn, 2010). Particularly, group I mGluRs induce a specific form of synaptic plasticity leading to long-term depression (LTD) of excitatory synaptic strength (mGluR LTD). In this work, we have observed that SOCE inhibition caused a strong impairment of DHPG-LTD in primary cortical neurons. In relation with SOCE components, STIM1 has been found to control mGluR1-dependent slow excitatory potentials in Purkinje neurons through its action in maintaining ER-Ca^2+^ levels (Hartmann et al., 2014), and both STIM2 knockout (Yap et al., 2017) and STIM1/STIM2 double knockout mice (Garcia-Alvarez et al., 2015b) have impaired LTP and LTD.

mGluR-LTD has been broadly studied in hippocampus and cerebellum (Jörntell and Hansel, 2006; Gladding et al., 2009; Lüscher and Huber, 2010), but less is known about other brain regions. It is still debated whether the expression mechanism of DHPG-LTD relies on presynaptic (neurotransmitter release) or postsynaptic (AMPA receptor internalization) changes (Anwyl, 2006). Moreover, in hippocampal neurons, the expression mechanism of DHPG-LTD depends on the developmental state of the synapse, being presynaptic in immature neurons but postsynaptic after neuronal maturation (Nosyreva and Huber, 2005). Our results show a clear reduction in the amplitude, but not frequency, of mEPSCs 15 min after DHPG application, which suggests a postsynaptic mechanism of expression. This agrees with the high maturation state of our neuronal cultures which has been previously documented when the BrainPhys Neuronal Medium was described (Bardy et al., 2015).

In cortical neurons, the strong impairment of DHPG-LTD and the failure to maintain DHPG-driven cytosolic Ca^2+^ signals in the presence of the SOCE blocker YM-58483 suggest that SOCE-mediated Ca^2+^ signal is essential to activate signaling pathways downstream mGluRs stimulation. It is particularly interesting that SOCE was required specifically for the maintenance of the calcium signal after mGluR activation, suggesting that this sustained signal is responsible for the long-term synaptic effects. SOCE may be acting directly by extracellular calcium influx through SOCs channels or by SOCE’s known function in maintaining the ER-Ca^2+^ levels (Samtleben et al., 2015), which are reduced by mGluRs stimulation. According to this, it has been demonstrated that ER-Ca^2+^ release from internal stores via IP_3_ generation after mGluR stimulation is required to induced LTD in cerebellum (Miyata et al., 2000; Kano et al., 2008). In the perirhinal cortex the induction of DHPG-LTD depends on interactions between the neuronal Ca^2+^ sensor protein (NCS-1) and protein interacting with C kinase (PICK1) in a Ca^2+^-dependent manner (Jo et al., 2008). However, it is relevant to note that the molecular mechanisms seem to differ between brain regions. Indeed, in hippocampus, DHPG-LTD is considered to be independent of PLC/IP_3_ pathway and postsynaptic Ca^2+^ (Schnabel et al., 1999; Fitzjohn et al., 2001; Mockett et al., 2011; Kim et al., 2015), but there is also evidence of IP_3_ mediated ER-Ca^2+^ release from dendritic spines and calmodulin activation for group I mGluR LTD (Holbro et al., 2009; Sethna et al., 2016).

Although Ca^2+^ influx through SOCs channels has been implicated in several functions in dendritic spines (Brini et al., 2014; Sun et al., 2014; Korkotian and Segal, 2017; Korkotian et al., 2017), SOCE proteins may also play roles outside the canonical SOCE pathway. A SOCE-independent activation of STIM proteins was first described in non-excitable cells (Lefkimmiatis et al., 2009; Tian et al., 2012; Shinde et al., 2013), and recently, this behavior has been also found in neurons. It has been shown that, in hippocampal neurons, STIM2 mediated PKA-dependent phosphorylation and trafficking of AMPA receptors is associated with an increase in cAMP levels (Garcia-Alvarez et al., 2015a). A recent study found that STIM1 overexpression in hippocampus impairs both synaptically and chemically induced mGluR-LTD, without affecting SOCE, among other calcium parameters (Majewski et al., 2017). As to the SOCE blocker used in this study to perform the experiments of DHPG-LTD, YM-58483, it seems to exert its action on the channel itself, without interfering with STIM proteins (He et al., 2005). Therefore, our results suggest a relevant role of Ca^2+^ entry through SOC channels themselves in DHPG-LTD.

In conclusion, our findings reveal a close relationship between mGluRs and SOCE in cortical neurons, and show that SOCE activation is a necessary step in the development of DHPG-driven cytosolic Ca^2+^ signals. Blocking SOCE activation upon mGluR stimulation prevented DHPG-LTD. Altogether the results suggest that, contrary to what has been observed in hippocampus, in cortical neurons SOCE-mediated Ca^2+^ signal could be relevant in generating downstream effects to evoke mGluR-LTD.

## Author Contributions

PG-S: designed and performed the experiments, analyzed the data and wrote the manuscript. AdA: designed and performed the experiments. JAE and JS: designed the study and wrote the manuscript. All authors reviewed the manuscript.

## Conflict of Interest Statement

The authors declare that the research was conducted in the absence of any commercial or financial relationships that could be construed as a potential conflict of interest.

## Footnotes

^1^https://lifescience.roche.com/en_es/brands/universal-probe-library.html
